# Glycobiology of Neuroblastoma: Impact on Tumor Behavior, Prognosis, and Therapeutic Strategies

**DOI:** 10.3389/fonc.2014.00114

**Published:** 2014-05-23

**Authors:** Nora Berois, Eduardo Osinaga

**Affiliations:** ^1^Laboratorio de Glicobiología e Inmunología Tumoral, Institut Pasteur de Montevideo, Montevideo, Uruguay; ^2^Departamento de Inmunobiología, Facultad de Medicina, Universidad de la República, Montevideo, Uruguay

**Keywords:** neuroblastoma, glycosylation, gangliosides, polysialic acid, galectin-1, glycosyltransferases, immunotherapy

## Abstract

Neuroblastoma (NB), accounting for 10% of childhood cancers, exhibits aberrant cell-surface glycosylation patterns. There is evidence that changes in glycolipids and protein glycosylation pathways are associated to NB biological behavior. Polysialic acid (PSA) interferes with cellular adhesion, and correlates with NB progression and poor prognosis, as well as the expression of sialyltransferase STX, the key enzyme responsible for PSA synthesis. Galectin-1 and gangliosides, overexpressed and actively shedded by tumor cells, can modulate normal cells present in the tumor microenvironment, favoring angiogenesis and immunological escape. Different glycosyltransferases are emerging as tumor markers and potential molecular targets. Immunotherapy targeting disialoganglioside GD2 rises as an important treatment option. One anti-GD2 antibody (ch14.18), combined with IL-2 and GM-CSF, significantly improves survival for high-risk NB patients. This review summarizes our current knowledge on NB glycobiology, highlighting the molecular basis by which carbohydrates and protein–carbohydrate interactions impact on biological behavior and patient clinical outcome.

## Introduction

Neuroblastoma (NB), the most common type of solid extra-cranial tumor in children, accounts for nearly 15% of pediatric cancer-related deaths (1). It is a very complex disease, extremely heterogeneous (2), able to regress spontaneously, even without therapy, but frequently displaying very aggressive behavior, refractory to current intensive multimodal therapy. Although over the past decade advances in NB staging through identification of molecular events responsible for different clinical behavior have improved risk stratification, not enough is known about how the features of this disease relate to its underlying biology and how this can be exploited to improve clinical outcome (3).

Clinical presentation of NB depends on primary tumor localization, most of them occur within the abdomen. Approximately half of the patients present localized forms of the disease, but dissemination occurs through lymphatic and hematogenous pathways, and about 35% have regional lymph node spread at the time of diagnosis. Bone, bone marrow, and liver are the most common sites of hematogenous spread. Most NB are undifferentiated tumors, which is an important issue in outcome prediction (4). The most common focal genetic lesion in NB is *MYCN* (V-myc myelocytomatosis viral-related oncogene) amplification, which occurs in approximately 22% of the cases and has been largely associated with poor outcome (2). However, among patients with *MYCN*-amplified low-stage NB, the outcome was significantly better for patients with hyperdiploid tumors when compared to those with diploid tumors (5), suggesting that tumor-cell ploidy could potentially improve risk classification. Another genetic disorder frequently found in NB is allelic loss of 11q. Although it seems mutually exclusive with *MYCN* amplification, it is frequently associated to other genetic abnormalities and poor clinical outcome (6). Pediatric oncologists classically distinguished between two risk-groups: (1) The low-risk group, consisting of non-*MYCN*-amplified localized tumors or the metastatic form in children younger than 18 months [survival rate of up to 90%, (7)]. (2) The high-risk group, comprising all *MYCN*-amplified NB, regardless of stage and age of the child, plus non-*MYCN*-amplified disseminated NB for children older than 18 months, usually very aggressive tumors that more frequently lead to death (8). However, relapse for low-risk patients constitutes a current concern (9), hence the International Neuroblastoma Risk Group Staging System (INRGSS) has recently established a new classification based on clinical criteria and image-defined risk factors (10). It distinguishes localized stages L1 and L2, and stages M and MS as disseminated forms. Based on this classification, age at diagnosis, histology and grade of tumor differentiation, *MYCN* status, presence/absence of 11q aberrations, and tumor-cell ploidy, NB patients can be sorted into very low-, low-, intermediate-, and high-risk groups according to percentage of 5 years disease-free survival (11). This classification will require validation in prospective clinical studies and solving some limitations as primary tumor dimensions using anatomic imaging, definitions of metastatic site, response not measurable by anatomical imaging (bone and bone marrow), as well as metastatic disease assessment using 123I-MIBG imaging and quantification of bone marrow disease (12).

## Gangliosides

Tumor cells, particularly tumors of neuroectodermal cell origin, express high levels of gangliosides (13). Besides their expression on tumor-cell membranes, gangliosides are also shed in the tumor microenvironment and eventually circulate in the patients’ bloodstream. These molecules are recognized to have multiple effects; for example, acting as cell-surface receptors and markers, participating in intercellular communication, and modulating cell signaling, cell cycling, and cell motility (14, 15). They have been implicated in the biology of various cellular processes, and linked to the behavior of many types of tumors (16). In NB, ganglioside composition is linked to biological and clinical behavior.

**Figure 1:**
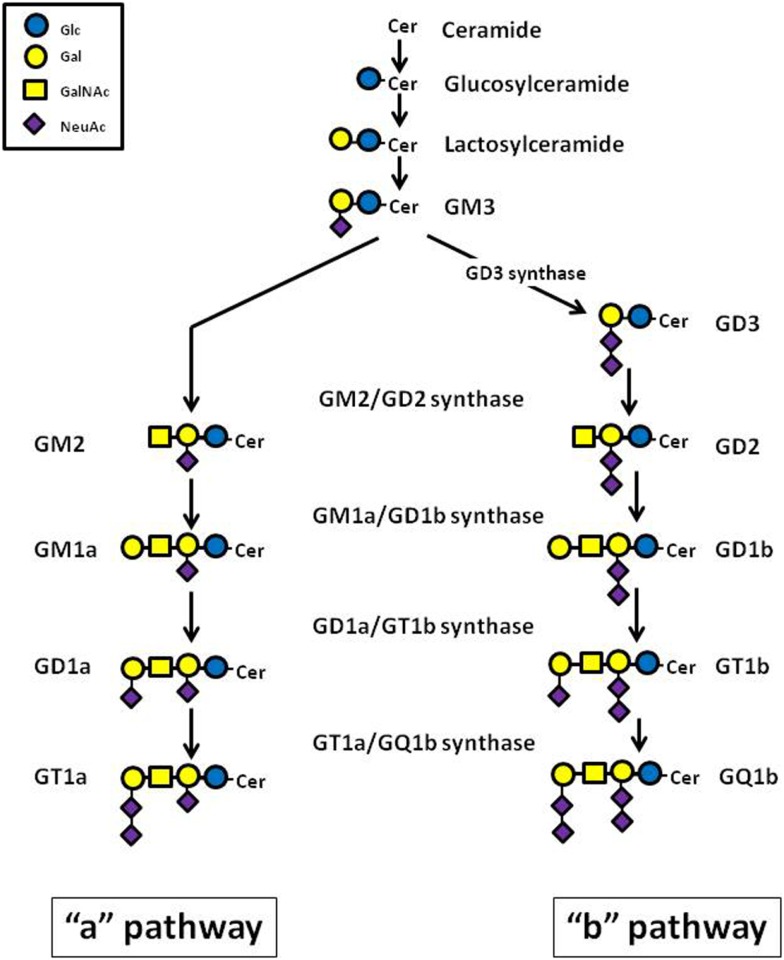
**Schematic representation of the major ganglioside biosynthesis pathways**.

Gangliosides consist of a carbohydrate chain, containing one or several sialic acid residues, and a lipid portion (ceramide backbone), which anchors the ganglioside molecule to the cell membrane (17). Ganglioside biosynthesis occurs in a sequential order of glycosylations via two major pathways designated as “a” (GM2, GM1a, and GD1a) and “b” (GD3, GD2, GD1b, GT1b, and GQ1b), from a common precursor (GM3) (Figure 1). Each ganglioside is structurally more complex than its precursor molecule, and the stepwise addition of monosaccharide or sialic acid residues in the Golgi apparatus is catalyzed by the same specific membrane-bound glycosyltransferases in both pathways (18) (Figure 1). Gangliosides can also be grouped into structurally simple (SG) and complex (CG) molecules. The enzyme GM1a/GD1b synthase (UDP-Gal:betaGlcNAc-beta-1,3-galactosyltransferase) converts its substrates, the simple gangliosides GM2 and GD2, into the corresponding initial complex ganglioside products, GM1a and GD1b (Figure 1). The key role played by this enzyme in human NB was confirmed by inducing high expression of GM1a/GD1b synthase in IMR-32 cells, which normally contain predominantly simple gangliosides, observing a rise of complex ganglioside expression, associated with reduced levels of simple gangliosides (19).

Ganglioside metabolism differs between NB tumors with different malignant potential, and may ultimately affect clinical behavior and patient outcome. It was observed that high levels of gangliosides of the “b” pathway (GD3, GD2, GD1b, GT1b, GQ1b) are predominant in infant NB compared to the same disease in older children (20). Evidence supports a role of some tumor gangliosides as prognostic indicators in NB. It is very interesting that low (≤35%) or absent expression of gangliosides of the complex “b” (CbG) pathway (GD1b, GT1b, and GQ1b) correlates with an aggressive biological phenotype in human NB tumors (21). This observation is consistent with reports in which a decreased or absent expression of two CbG subspecies, GD1b and GT1b, was linked to reduced survival in NB patients (22, 23). High expression of complex gangliosides, both complex “a” gangliosides (CaG) and CbG, has been shown to inhibit aggressive tumor-cell behavior *in vitro* (e.g., cellular proliferation and migration) and to enhance differentiation (24, 25). In this context, complex gangliosides have been proposed as useful biomarkers to predict clinical outcome, to stratify patients with NB for purposes of tailoring anti-cancer treatment, or to monitor effectiveness of treatment.

Retinoic acid is successfully used in maintenance therapy of disseminated NB (26). Treatment with this pharmacological agent induces a dramatic shift from synthesis of simple gangliosides toward predominant expression of structurally complex “a” and “b” pathway ganglioside molecules in some NB cell lines (27). Predominant expression of complex gangliosides can be considered a biochemical marker of increasing neuronal differentiation. The retinoic acid-induced rise of CbG expression in NB cells represents a transition into a ganglioside pattern associated with clinically less-aggressive NB tumors. These authors demonstrated that treatment with retinoic acid markedly enhances the activity of GD1b/GM1a synthase, resulting in increased expression of the complex gangliosides in NB cell lines.

When compared with normal brain tissue, NB tumors were found to overexpress the disialoganglioside GD2 (“b” pathway) (28). GD2 is a surface glycolipid antigen normally found on neurons, peripheral nerve fibers, and skin melanocytes. In NB, GD2 is expressed homogeneously and abundantly in virtually all neuroblasts and facilitates the attachment of tumor cells to the extracellular matrix (29, 30). Because of the widespread expression of GD2 in NB tissue, contrasting with the more benign tumors ganglioneuroma and ganglioneuroblastoma, GD2 is a sensitive diagnostic marker which can help to discriminate NB from other related tumors (28, 31). Expression of GD2 is an indicator of the presence of NB, and high levels of circulating GD2 have been correlated with a more rapid disease progression among patients in advanced stages of the disease (32). However, patient outcome was independent of GD2 expression in tumor tissues (21), suggesting that GD2 is useful for diagnostic purposes but not for prognostic ones. Surface GD2 antigen is an important molecular target for NB therapy and specific monoclonal antibodies have emerged as a major therapeutic development for high-risk NB patients (see below).

Gangliosides overexpressed and actively shedded by tumor cells can modulate the function of normal cells present in the tumor microenvironment (33). Several studies have demonstrated modulation of growth factor signaling pathways through the epidermal growth factor (EGF), fibroblast growth factor (FGF), platelet-derived growth factor (PDGF), Trk family, and insulin receptors (15, 34, 35). It was also found that the enrichment of human umbilical vein endothelial cell (HUVEC) membranes with purified GD1a ganglioside results in amplified VEGF-induced signaling and the associated cellular responses of proliferation and migration, important for angiogenesis (36). In addition, tumor gangliosides induce robust murine tumor angiogenesis *in vivo* (37). Also, tumor-derived gangliosides may promote tumor development by suppressing immune cell function and promoting immune evasion mechanisms (38). Early studies found that gangliosides, predominantly GD2, isolated from the human NB cell line LAN5, inhibit murine cellular immune responses *in vivo* (39, 40). The mechanisms by which gangliosides suppress tumor immunity, although not fully understood, involve the regulation of different immune cells. Tumor-derived gangliosides can inhibit tumor-specific cellular cytotoxicity, suppressing the lytic activity of CD8+ T-cells (41), as well as NK cell-mediated cytotoxicity in a Siglec-7-dependent manner (42). Dendritic cell (DC) development and function is critical in the initiation phase of any antigen-specific immune response against tumors. There is an important body of evidence indicating that NB-derived gangliosides regulate development of tumor immunity through the inhibition of DC function (43). Shurin et al. demonstrated that NB gangliosides inhibit the generation of functionally active DCs, playing a role in tumor-induced immunosuppression (44). NB gangliosides may induce inhibition of DC function causing CD40 signaling deficiency (45) and alterations in TLR signaling (46). Gangliosides promote DC population development characterized by decreased CD86 expression (costimulatory signal), and decreased interleukin-12 and interleukin-6 production. When these cells are used as antigen-presenting cells, CD4 T-cells are primed to proliferate normally, but have a defect in T helper (Th) effector cell development. This defect in Th effector cell responses is associated with the development of regulatory T-cell activity that can suppress the activation of previously primed Th effector cells in a contact-dependent manner (47). In total, these data suggest that ganglioside-exposed DC promote regulatory T-cell activity that may have long-lasting effects on the development of tumor-specific immune responses.

## *N*- and *O*-Protein Glycosylation

*N*-linked glycosylation is a highly regulated post-translational modification, which is involved in several biological processes such as protein folding and conformation, oligomerization, cell–cell interactions, and targeting proteins to sub- or extracellular locations. It was found that intercellular adhesion molecule-2 (ICAM-2) completely suppressed disseminated tumor development *in vivo* in a murine model of metastatic NB (48). The authors also observed that ICAM-2 suppressed NB cell motility and growth in soft agar *in vitro*. These effects on NB cells depended on the interaction of ICAM-2 with the cytoskeletal linker protein α-actinin. ICAM-2 has six *N*-linked glycosylation sites at asparagines 47, 82, 105, 153, 178, and 187. The substitution, using site-directed mutagenesis, of asparagine by alanine at glycosylation sites, was found to reduce *N*-glycosylated ICAM-2, displaying a significantly attenuated ability to suppress metastatic properties of NB cells (49). Anaplastic lymphoma kinase (ALK) has been identified as a major NB predisposing gene, and activating mutations have also been identified in a subset of sporadic NB tumors (50). ALK protein expression is significantly up-regulated in advanced/metastatic NB, and overexpression of either mutated or wild-type ALK, defines poor prognosis patients (51). Inhibition of ALK activity in NB cell lines has already been approached by using specific small molecules (51, 52). ALK has 16 highly conserved putative sites of *N*-linked glycosylation in the extracellular domain. It was demonstrated that inhibition of *N*-linked glycosylation impairs ALK phosphorylation and disrupts downstream pro-survival signaling, as well as cell viability, in NB cell lines harboring mutated or amplified ALK (53), suggesting that inhibition of this post-translational modification could be a promising therapeutic approach.

Cell-surface mucins are glycoproteins carrying large numbers of *O*-linked oligosaccharides. The most abundant form of *O*-linked glycosylation in higher eukaryotes, termed “mucin-type,” is initiated by the covalent linkage of an α-*N*-acetylgalactosamine residue (GalNAc) to the hydroxyl group of Ser/Thr residues, catalyzed by UDP-GalNAc:polypeptide-*N*-acetyl-galactosaminyl-transferases (GalNAc-T). GalNAc-T is a complex family of up to 20 isoenzymes characterized to date (54). Altered *O*-glycan profile is a hallmark of carcinomas, which expresses truncated *O*-glycosylated tumor-associated antigens such as Tn, sialyl-Tn, and TF (55). In contrast, the complete absence of these antigens was reported in NB (13, 56). However, some evidence suggests that *O*-glycosylation pathways could play an important role in NB biology. Our research group has demonstrated that GalNAc-T9 and GalNAc-T13 might be useful tumor markers associated with low or high tumor aggressiveness, respectively (57, 58) (see below). In addition, the better outcome of NB patients is also predicted by β1,3-*N*-acetylglucosaminyltransferase-3 (B3GNT3) expression, the enzyme responsible for extended core 1 *O*-glycan (T antigen) oligosaccharide synthesis (59) (see below).

## Polysialic Acid

Polysialic acid (PSA) is a developmentally regulated linear homopolymer of α-2-8-linked sialic acid (up to 200 residues long) located on the outer chains of *N*-linked oligosaccharides (60, 61). This unique glycosylation is attached mainly to the neural cell-adhesion molecule (NCAM) (62), which is expressed on cells of neuroectodermal origin and plays a pivotal role in neural tissue development and regeneration. It is well documented that the presence of the highly negatively charged PSA on NCAM reduces NCAM-mediated adhesion processes as well as NCAM-independent cell interactions, such as cadherin-mediated cell-adhesion (63, 64). There are several isoforms of NCAM due to different sizes, three of which can carry PSA: NCAM-180 and NCAM-140 (integral membrane isoforms), and NCAM-120 (isoform anchored to the plasma membrane via a glycosyl phosphoinositol) (65, 66). The mRNA encoding the 120 kDa protein species was the most abundant isoform found in the adult brain, ganglioneuromas and ganglioneuroblastomas, but the mRNA encoding the 180 kDa species was predominant in NB (67). The polysialylated form of NCAM (PSA-NCAM) is widely expressed in embryos, especially in the brain, where it augments cellular motility and assists neuron outgrowth (68). Conversely, PSA-NCAM is not present in the adult brain, other than in areas requiring synaptic regrowth, such as the hippocampus and olfactory bulb (69, 70).

Although PSA is virtually absent in most adult tissues, it is re-expressed during the progression of some malignant tumors, such as NB, rhabdomyosarcoma, non-small cell lung cancer (NSCLC), and small cell lung cancer (SCLC) (71–75). High PSA-NCAM levels have been correlated with malignant potential and poor prognosis of SCLC, NB, glioblastoma, medulloblastoma, and rhabdomyosarcoma (73, 76–81). PSA promotes tumor-cell migration *in vitro* and affects tumor-cell differentiation by attenuating NCAM signaling (82). It has been reported that PSA increases the motility of SCLC cells and allows cancer cells to detach from the primary tumor, thus favoring the formation of metastatic foci (76). Regarding NB, PSA has a direct impact on tumor-cell growth. The presence of PSA-NCAM has been shown to increase proliferative cell activity *in vitro* (83). PSA plays an important role as regulator of NB cell migration. It was demonstrated that the migratory effect is NCAM-dependent, but independent of FGF receptor activity (84). Removal of PSA from the cell-surface led to reduced proliferation and neuronal differentiation (82). *In vivo* studies indicate that PSA-NCAM reduces the adhesiveness of tumor cells and promotes dissemination, and its expression is also closely associated with tumor invasion and metastasis (85). In this work, five human NB cell lines (three of which PSA-NCAM-expressing) were xenografted into SCID mice. Disseminated lung micrometastases developed from the three PSA-expressing xenografts, but not from the negative-expressing tumors. In clinical tissues, PSA-NCAM expression is indicative of undifferentiated NB, correlating with aggressive advanced disease (79, 86, 87). Its expression also correlates with N-MYC amplification (86) and its serum detection is a disease marker (78).

Two polysialyltransferases, ST8SiaII (STX) and ST8SiaIV (PST) have been shown to synthesize PSA independently. While ST8SiaII is expressed predominantly during embryonic development, ST8SiaIV is the major polysialyltransferase in the adult brain (88). Polysialylation is a protein-specific reaction in which the polysialyltransferases initially recognize a protein sequence prior to effecting glycan modifications (89, 90). STX appears to specifically polysialylate NCAM, whereas PST may additionally polysialylate other glycoproteins (91). STX is the prominent enzyme in tumor cells (73, 92, 93). In a panel of NB cell lines, co-expression of PST and STX was observed, but STX levels were significantly higher than PST in most of them (94). In NB tumors, STX expression is consistently high (93) (see below). The fact that PSA promotes a more aggressive behavior in cancer cells and that STX (absent from the adult brain) is the dominant polysialyltransferase in tumors which express PSA, strongly suggests that STX could be a good therapeutic target to inhibit tumor invasion and metastasis (95). The cytidine monophosphate (CMP) is a compound capable of reducing STX-mediated polysialylation of NCAM (96). In addition, migration of IMR-32 NB cells decreased after application of the sialic acid precursor ManNProp, which interferes with polysialylation (97). However, despite the inhibitory effects of CMP and *N*-acyl mannosamines on PSA synthesis, they are unlikely therapeutic agents due to their unfavorable physicochemical properties. They are hydrophilic carbohydrate derivatives which lack the ability to efficiently penetrate cell membranes. Inhibitors with properties that facilitate cell penetration to the Golgi apparatus (the location of the STX) and possess robust drug metabolism and pharmacokinetic properties are still to be discovered (95).

## Galectin-1 is Associated with NB Aggressiveness

Lectin–glycan interactions play a fundamental role regulating mechanisms implicated in tumor immune escape (98). Galectins are a family of carbohydrate-binding proteins that share a carbohydrate recognition domain for β-galactosides and are involved in cell adhesion, migration, differentiation, angiogenesis, proliferation, mRNA splicing, and apoptosis (99). Their function takes place both extracellularly, by interacting with cell-surface and extracellular matrix glycoproteins and glycolipids, as well as intracellularly, by interacting with cytoplasmic and nuclear proteins to modulate signaling pathways. Current research indicates that galectins play an important role in cancer; they contribute to neoplastic transformation, tumor-cell survival, angiogenesis, and tumor metastasis (100). In NB, galectin-1 (Gal-1) has emerged as an interesting target. It regulates complex signaling pathways involved in tumor–host interaction (101) and angiogenesis (102). Tumor secretion of Gal-1 contributes to the immunosuppressive potential of a wide range of tumors by limiting T-cell survival and impairing DC function (103–105). NB tumors, particularly those with poor prognosis, express high levels of Gal-1 (106). The neurotrophin receptor TrkB is also expressed in NB with poor prognosis, conferring invasive and metastatic potential to the tumor cells as well as enhancing therapy resistance. Blocking Gal-1 function strongly reduced the migratory and invasive capacities of TrkB expressing NB cells (106). Soluble Gal-1 was found to be actively secreted by different NB cell lines (107). The authors found that cancer-derived Gal-1 modulates T-cell and DC, resulting in increased tumor growth and metastasis. Interestingly, Gal-1 blockage efficiently inhibits primary tumor growth and metastasis, strongly suggesting that Gal-1 could be a therapeutic target in NB. Similarly, inhibition of Gal-1 function resulted in tumor rejection in other animal models (108, 109).

## Glycosyltransferases as Tumor Markers in NB Patients

Clinical outcome for high-risk NB patients remains poor in spite of multimodal treatment including chemoradiotherapy, surgery, and stem cell transplantation. Because osteomedullary recurrences are frequent in patients with NB, the development of sensitive and specific methods to detect rare tumor cells in bone marrow or peripheral blood is important, both for risk assessment at diagnosis and for evaluating response to therapy. Conventional cytology, traditionally used for occult disseminated disease detection, has limited sensitivity and was improved by immunological strategies (110). However, the effectiveness of cell-surface antigen detection largely depends on epitope exposure and accessibility to antibodies, so techniques based on amplified mRNA detection have demonstrated a higher sensitivity for this purpose (111).

As stated above, cancer hallmark aberrant glycosylation is associated with differential expression of antigens as well as some enzymes, such as glycosyltransferases and glycosidases. In the personalized approach to cancer diagnosis and treatment, these enzymes are proving to have clinical relevance as cancer biomarkers for different tumors (112). In this study, we show results reported for NB patients (Table 1). GD2 synthase (β1,4-*N*-acetylgalactosaminyltransferase, EC 2.4.1.92), the key enzyme for GD2 synthesis, has been largely evaluated, both by immunological approaches as well as by nucleic acid techniques. The first monoclonal antibodies specific for this enzyme, which date from more than two decades ago, demonstrated a high specificity and sensibility of 0.01% for neuroblast detection in bone marrow samples (113). However, mRNA techniques (RT-PCR analysis) may further improve detection of rare NB cells, and highly sensitive and specific methods that measure GD2 synthase mRNA have been reported (111, 114). Cheung et al. communicated its clinical utility in evaluating adjuvant therapy in NB patients (115), although it seems less specific as a prognostic marker at diagnosis. In a comparative and prospective study of a consecutive patient cohort, Träger et al. analyzed the clinical significance of tyrosine hydroxylase (TH), dopa decarboxylase (DDC), and GD2 synthase transcripts (116). They found that high expression of TH and DDC, both in peripheral blood and bone marrow corresponds to metastatic NB at diagnosis, residual disease, and poor outcome, while GD2 is less specific at the mRNA level for NB detection. In the same way, the International Neuroblastoma Risk Group Task Force, in an effort to achieve a consensus on standard operating procedures in order to improve and standardize management of children with NB, agreed on the detection of rare neuroblasts in bone marrow, peripheral blood, and peripheral blood stem cells by immunocytochemistry using GD2 and by QRT-PCR for TH mRNA (117).

**Table 1:** **Glycosyltransferases as neuroblastoma (NB) tumor markers**.

Enzyme	Method/sample	Clinical significance	Reference
β1,4-*N*-acetylgalactosaminyltransferase (GD2 synthase)	ICC/bone marrow	Molecular marker of metastatic NB	(113)
	RT-PCR ECL/bone marrow	Molecular marker of metastatic NB	(114)
	RT-PCR ECL/bone marrow	Molecular marker of metastatic NB	(111)
	qRT-PCR/bone marrow	Marker for minimal residual disease	(115)
	qRT-PCR/bone marrow-PB	Prognostic marker (poor outcome)	(116)
Sialyltransferase STX (ST8SiaII)	qRT-PCR/bone marrow	Molecular marker of metastatic NB	(93)
*N*-acetylglucosaminyltransferase V (GnT-V)	qRT-PCR/primary tumor	Prognostic marker (better outcome)	(123)
UDP-polypeptide GalNAc-transferase 13 (GalNAc-T13 – *GALNT13*)	RT-PCR/bone marrow	Molecular marker of metastatic NB	(58)
UDP-polypeptide GalNAc-transferase 9 (GalNAc-T9 – *GALNT9*)	RT-PCR/primary tumor	Prognostic marker (better outcome)	(57)
β1,3-*N*-acetylglucosaminyltransferase-3 (*B3GNT3*)	IHC/primary tumor	Prognostic marker (better outcome)	(59)
β1,4-*N*-acetylgalactosaminyltransferase 3 (*B4GALNT3*)	IHC/primary tumor	Prognostic marker (better outcome)	(142)
	IHC/primary tumor	Prognostic marker (poor outcome)	(143)

*ICC, immunocytochemistry; RT-PCR, reverse transcriptase-polymerase chain reaction; ECL, electrochemiluminescence; PB, peripheral blood*.

However, despite the increased sensitivity of those methods, it is clear that not all patients with disseminated disease are detected, reflecting limitations in the procedures. This could be due to analysis of a small volume of sample and also to limitations as a consequence of tumor heterogeneity, which could possibly be solved by multi-marker assays. Therefore, research for novel specific markers is warranted and investigators are working in this direction. Cheung et al. hypothesize that sialyltransferase STX (ST8SiaII), the key enzyme for PSA synthesis, can potentially be a sensitive marker for metastatic NB (93). As stated above, PSA is critical for cellular adhesion, neuronal migration, and tumor metastasis, and it is highly expressed in many human cancers, including NB. Since the enzyme STX has restricted expression in postnatal tissues, including spleen and leukocytes, and because of its up-regulation during dedifferentiation in a number of human cancers, it could be a potential marker for metastatic tumor cells in blood or bone marrow. The authors optimized a quantitative RT-PCR in order to evaluate STX expression in tumor samples, cell lines, and bone marrow from a cohort of high-risk NB patients enrolled in a post-induction immunotherapy protocol utilizing anti-GD2 antibody 3F8. Patient follow-up demonstrated that STX positivity and its transcript level were highly prognostic for PFS (*p* < 0.0005) (93). However, like TH and GD2 synthase, the utility of STX as a single marker is constrained by the inherent heterogeneity of human NB, so its utility is expected to improve if combined into a marker panel.

*N*-acetylglucosaminyltransferase V (GnT-V) is one of the most relevant glycosyltransferases associated to tumor invasion and metastasis. Previous studies demonstrated that an increased amount of β1,6-branched oligosaccharides, formed by the action of GnT-V, is correlated with metastatic potential (118). This enzyme has been associated to tumor progression in human breast and colon neoplasia (119), and could be a prognostic marker in human colorectal carcinoma (120, 121). However, for lung cancer, Dosaka-Akita et al. reported that the lower expression of GnT-V is associated with shorter survival and poor prognosis (122). Regarding NB, assessing GnT-V mRNA expression by real-time PCR, a significant correlation was observed between higher expression levels and a favorable prognosis in a cohort of 126 patients (123). GnT-V-knockdown in NB cells showed a tendency to escape from retinoic acid-induced apoptosis, supporting the notion that a higher expression of GnT-V is correlated with a favorable prognosis of NB patients.

Novel techniques in marker discovery, such as genome-wide gene expression array analyses, have been successfully applied to NB, leading to identification of useful markers for minimal residual disease diagnosis (124) and prognostic markers (125, 126). Using the same strategy, we studied the transcriptome profiles of malignant neuroblasts established from the human *MYCN*-amplified IGR-N-91 model (127). Comparative gene expression profiles obtained with Agilent oligo microarrays, between primary tumor versus bone marrow metastatic cell lines, revealed a set of 107 differentially expressed genes in the metastatic neuroblasts (58). Up-regulated genes were involved in chemoresistance, cell motility, and neuronal structure/signaling, and surprisingly, the most strongly up-regulated gene was *GALNT13*, followed by *ABCB1*, whose higher expression has been previously described (127, 128) and highlights the fact that acquired drug resistance is an important cause of NB treatment failure. *GALNT13* encodes the UDP-GalNAc:polypeptide GalNAc-transferase-13 (GalNAc-T13), described as specifically expressed in neuronal tissue (129), belonging to the enzyme family which catalyzes the first step of mucin-type *O*-glycosylation (54). Our analysis of *GALNT13* mRNA transcripts in various NB cell lines, as well as in favorable and high-risk neuroblastic tumors, showed that *GALNT13* is highly expressed in more aggressive neuroblasts but is not correlated with *MYCN* amplification and/or expression. Looking for a potential marker for NB disseminated cells, bone marrow samples harvested at diagnosis of NB patients with different stages of the disease were analyzed for *GALNT13* expression, and compared to other proposed markers (TH, GD2 synthase, DDC, and conventional cytology). Overall survival by Kaplan–Meier analysis demonstrated the best correlation with a poor clinical outcome for *GALNT13* expression (58). We then proposed GalNAc-T13 as a new informative marker for the molecular diagnosis of BM involvement and the follow-up of minimal residual disease in NB patients. However, to elucidate the biological role of *GALNT13* in NB, further work is needed to analyze the molecular mechanisms that regulate this gene’s expression and to identify the enzyme acceptor substrates potentially involved in metastatic activity of these cells.

Performing a *GALNTs* expression profile in several NB cell lines, we observed that a few enzymes showed an opposite expression pattern in some of them. *GALNT9*, described by Toba et al. as restricted to the brain (130), was found expressed in neuroblasts derived from the primary tumor in the experimental model IGR-N-91, but not in neuroblasts derived from bone marrow metastases (57). The expression profile in different NB cell lines was restricted to substrate adherent (S)-type ones, exhibiting weaker tumorigenesis, invasiveness, and metastatic properties (131), while it was always negative in more aggressive neuronal (N)-type cell lines. When a tumor cohort from 122 NB patients was analyzed, *GALNT9* expression was associated with high overall survival, independently of the standard risk-stratification covariates, and it was significantly associated with disease-free survival for patients currently classified as low risk (57). *GALNT9* expression is probably a marker for more mature stages of neuroblastic tumor cells, which are associated with less aggressivity. This isoenzyme belongs to a subfamily among *GALNT* genes, together with *GALNT8, GALNT18*, and *GALNT19*, and exhibits significant sequence differences from the other members of the *GALNT* family, and also a low activity in a small number of peptides (54). This fact, together with the opposite expression of *GALNT13*, up-regulated in more aggressive neuroblasts, lead to the hypothesis that these enzymes could have biological relevance in NB behavior. They could be candidates for a panel of tumor markers to be included in large prospective cooperative multi-center studies, performed according to the INRG standard operating procedures (117).

Another interesting glycosyltransferase, β1,3-*N*-acetylglucos aminyltransferase-3 (*B3GNT3*), is a member of the β3GlcNAc-T family, composed of at least eight isoenzymes, and responsible for adding GlcNAc to core 1 (T antigen) in a β1,3 linkage, forming extended core 1 oligosaccharides (132). This enzyme is expressed in lymphocytes and neutrophils, and could contribute to the expression of core 1-derived *O*-glycans, which play an essential role in E-selectin adhesion (133). It has been demonstrated, in a colon cancer experimental model, that E- and P-selectin are essential protagonists in cell–cell adhesion by means of glycosylated ligands in tumor cell-surface, which are responsible for lung metastatic development (134). Gakhar et al. suggested a similar mechanism as responsible for the attachment of glycoproteins on the surface of prostate cancer circulating tumor cells, acting as functional ligands for E-selectin expressed on endothelial cells (135). However, contrary to what one might expect, in NB, *B3GNT3* expression has been correlated with favorable clinical outcome (59). Increased *B3GNT3* expression in NB tumor tissues correlated well with the histological grade of differentiation, while undifferentiated tumors remain more frequently negative. Univariate and multivariate analyses revealed that positive *B3GNT3* expression in tumor tissues predicted a better survival of NB patients, independently of other prognostic markers (59). In the same work, cell line experiments expressing and knocking down the enzyme demonstrated that *B3GNT3* expression significantly correlated with suppression of core 1 expression, as well as malignant phenotypes including migration and invasion.

β1,4-*N*-acetylgalactosaminyltransferase III (*B4GALNT3*) cloned by Sato et al., has been described as expressed in stomach, colon, and testis (136). This enzyme can transfer GalNAc residues to non-reducing terminal GlcNAc-β leading the synthesis of GalNAcβ1–4GlcNAc (also known as LacdiNAc or LDN), which is a unique terminal structure in the outer chain moieties of human *N*-glycans (137), and also in *O*-linked oligosaccharide structures (138). The largest amount of *B4GALNT3* transcripts were found in gastric tissues, followed by colon, testis, and adrenal glands (136). Gastric expression of *B4GALNT3* was found regulated by cellular differentiation (139). In the human colon, Huang et al. reported that *B4GALNT3* is up-regulated in primary tumors comparing with the normal mucosa (140). They performed *in vitro* and *in vivo* experiments showing that overexpression of this enzyme increases malignant phenotype of colon cancer cells, and these phenotypic changes are associated with enhanced integrin and mitogen-activated protein kinase (MAPK) signaling, suggesting that *B4GALNT3* may play a crucial role in promoting malignant behavior of colon cancer (140). However, the same research team has recently published that *B4GALNT3* overexpression in colorectal cancer cells suppressed cell migration, invasion, and adhesion, while *B4GALNT3* knockdown enhanced malignant cell phenotypes promoting cell migration and invasion (141). Surprisingly, a similar situation was found for NB. Firstly, Hsu et al. communicated that *B4GALNT3* expression positively correlates with the differentiation status of NB, predicting a favorable prognosis for patients and suppressing the malignant phenotype in cell lines experiments via decreasing β1-integrin signaling (142). However, opposite results were reported 2 years later by the same group and for the same cohort of patients, showing *B4GALNT3* expression in NB tumors correlating with advanced clinical stage, unfavorable histology, and lower survival rate (143). In face of this controversy, the role of *B4GALNT3* in NB is not clear, and further work is necessary to elucidate the real clinical significance of the enzyme in tumor behavior and subjacent molecular mechanisms.

## GD2, a Good Target for NB Immunotherapy

The choice of NB treatment depends on patient risk group stratification. While some carefully selected cases with favorable features could benefit from observation alone (144), usually high-risk patients are subject to multimodal aggressive therapeutic plans (including surgery, chemotherapy, radiotherapy, and immunotherapy) (145). Clinical outcome for high-risk groups remains poor due to frequent recurrence of osteomedullary disease. The identification of newer tumor targets and immunotherapy strategies may generate novel therapeutic approaches which could combine to improve survival and cure rates. Rational approaches to target ALK and MYCN, which arise from the two most common and potentially significant genetic alterations in NB, are emerging and we must expect that some therapeutic options will become available in the near future (146).

Immunotherapy is intended to redirect the immune system to target tumors and tumor-associated antigens, leading to the elimination of malignant cells. Multiple forms of immunotherapy are being used in clinics, including monoclonal antibodies targeting tumor cell-surface antigens or disrupting the normal checkpoints that inhibit anti-tumor immune responses, cytokines that modify innate or adaptive immunity, tumor vaccines, as well as adoptive cellular therapies (147–151). There is increasing data regarding more efficient immunotherapeutic protocols designed based on cancer-associated glycan structures (152). NB provides an attractive target for immunotherapy as it expresses certain glycan antigens not widely detected in non-embryonic tissues, such as GD2 and *O*-acetyl GD2 (153). In fact, the National Cancer Institute pilot program for the prioritization of the most important cancer antigens ranks GD2 as 12 out of 75 potential targets for cancer therapy (154). Anti-GD2 antibodies have been actively tested over the past two decades in clinical trials for NB, and have emerged as a major therapeutic development for high-risk cases, with proven safety and efficacy (155, 156). In particular, four anti-GD2 antibodies (3F8, hu3F8, ch14.18, and hu14.18) have been extensively tested in clinics (157). The murine IgG3 monoclonal antibody (MAb) 3F8 was the first well-characterized anti-GD2 antibody (158). Its efficacy in treating NB was initially described in 1987 in the report of a Phase 1 trial which included patients with refractory high-risk NB (159). MAb 3F8 mediates highly efficient antibody-dependent cell-mediated cytotoxicity (ADCC) of NB in the presence of human natural killer (NK) cells and granulocytes *in vitro* (160–162). Moreover, it induces complement-mediated cytotoxicity (CMC), because NB cells lack decay-accelerating factor CD55 (163) and homologous restriction factor CD59 (164). IL-2 and GM-CSF were shown to enhance ADCC *in vitro* by activating cytotoxic NK cells and neutrophils (160, 165, 166). When combined with the cytokine GM-CSF and 13-cis-retinoic acid, 3F8 induced >60% long-term survival among high-risk patients with metastatic disease, treated at first remission (167). MAb 3F8 was recently humanized (hu3F8) (168) and is currently in Phase I trials (157). MAb 14.G2a is an IgG2a class switch variant of MAb 14.18, originally isolated as an IgG3 isotype (169). 14.G2a showed higher *in vitro* and *in vivo* ADCC than 14.18 and was subsequently modified for clinical development. MAb 14.G2a was chimerized to form ch14.18 and humanized to form hu14.18 (named after the original mouse 14.18 IgG3 isotype) (170). A phase III randomized trial showed that ch14.18, when combined with GM-CSF and interleukin-2, in high-risk NB patients, was associated with significantly improved survival compared to standard therapy after a 2-year follow-up period (171).

Several approaches have been developed to enhance anti-tumor efficacy of anti-GD2 monoclonal antibodies and fragments: immunocytokines (172), immunotoxins (173), antibody drug conjugates (174), radiolabeled antibodies (175), targeted nanoparticles (176), T-cell engaging bispecific antibodies, and chimeric antigen receptors (CARs) (157). Although the CAR technology is still at an early stage, clinical trials have already shown significant anti-tumor activity in NB patients. In this approach, antibody and cell-based immunotherapy of cancer has converged in the development of engineered T-cells which express the antigen binding site of a MAb (commonly an antibody-derived single-chain fragment) coupled with the intracellular signaling portion of the T-cell receptor (TCR) (177, 178). A key advantage is that CARs target native, rather than processed antigens; consequently, their function is not hampered by HLA downregulation, frequently observed in human cancer (179). CAR-T-cells targeting cancer-associated ganglioside antigens such as GD2 (180) or GD3 (181) have been developed. A chimeric GD2-specific receptor on T lymphocytes exhibited *in vitro* anti-melanoma activity and increased survival of mice xenografted with a human melanoma cell line (182). Cytotoxic T lymphocytes (CTLs) expressing a chimeric GD2-specific receptor were generated using the Epstein-Barr virus (183). Infusion of these genetically modified cells (CAR-CTL anti-GD2) was associated with tumor regression or necrosis in half of the tested patients (183). After that, Louis et al. reported complete remission of 3 out of 11 patients with active disease treated with CAR-CTL anti-GD2 infusions, on a long-term clinical and immunologic follow-up (184).

## Concluding Remarks

**Figure 2:**
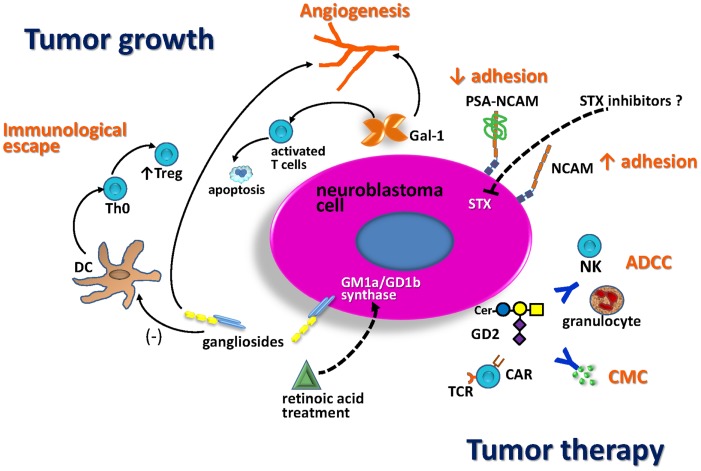
**Schematic representation of NB glycobiology impact on tumor growth and anti-tumor therapy**. Treatment with retinoic acid markedly enhances the activity of GD1b/GM1a synthase, resulting in increased expression of complex gangliosides, associated with less-aggressive tumors. NB gangliosides promote dendritic cell (DC) to develop with decreased costimulatory signals and IL-12 production. These DC promote differentiation of Th0 cells toward regulatory T-cells (Treg). Gal-1 secreted by NB also contributes to the immunosuppressive tumor microenvironment, limiting T-cell survival and impairing DC function. Both, gangliosides and Gal-1 contribute to tumor angiogenesis. The presence of polysialic acid (PSA) on NCAM reduces NCAM-mediated adhesion processes promoting NB cell migration. The fact that STX is the dominant polysialyltransferase for PSA biosynthesis in NB suggests that this enzyme could be a good therapeutic target. GD2 is a relevant antigen for NB immunotherapy. Anti-tumor activity of anti-GD2 antibodies is mediated by antibody-dependent cell-mediated cytotoxicity (ADCC) in the presence of human natural killer (NK) cells and granulocytes, as well as by complement-mediated cytotoxicity (CMC). Anti-GD2 chimeric antigen receptor T-cells (CAR-T-cells) activity could induce NB tumor regression.

Different molecules involved in NB glycobiology play key roles in tumor growth and are potential targets for anti-tumor therapy (Figure 2). It is well-established that gangliosides and PSA-NCAM impact in the aggressiveness of NB cells as well as in the patients’ clinical outcome. Some evidence suggests that enzymes which catalyze *O*-glycan biosynthesis could play a role regulating NB behavior, but the molecular basis of these observations remain to be established. Some aspects of NB glycobiology do not only affect the tumor-cell phenotype (e.g., proliferation, differentiation, and adhesion), but also contribute to local microenvironment and immune response control. For example, NB-derived gangliosides inhibit the generation of functionally active DCs, leading to an increase of Treg cells inside the tumor, which suppress effective anti-tumor responses. In the same way, Gal-1 secretion by NB cells stimulates tumor growth by inducing local immunosuppressive activities and angiogenesis. This is relevant because Gal-1 has been characterized as a promising target for therapy in cancer models, using synthetic and natural inhibitors. Virtually all NB cells express ganglioside GD2. This is a relevant antigen for anti-tumor immunotherapy strategies because it is highly expressed in NB cell-surface, and the blood–brain barrier limits the side effects due to normal expression in neuronal cells. Anti-GD2 antibody (ch14.18) combined with IL-2 and GM-CSF represent the latest major therapeutic advance for high-risk NB in the last decade. This immunotherapy was demonstrated to be safe as well as a key component to achieve cure or long-term remission in patients with residual disease. In addition, recent clinical success has underscored the potential of NB immunotherapy based on the adoptive cell transfer of engineered T lymphocytes (CAR-CTL anti-GD2) in order to mediate strong and durable clinical responses. Several approaches may further enhance anti-tumor activity and persistence of circulating CAR-modified cells. Safe transfer of CAR-based immunotherapy into clinical practice could represent a potential alternative to conventional treatment options for NB patients.

## Conflict of Interest Statement

The authors declare that the research was conducted in the absence of any commercial or financial relationships that could be construed as a potential conflict of interest.
