# Corrigendum: Glycobiology of Neuroblastoma: Impact on Tumor Behavior, Prognosis, and Therapeutic Strategies

**DOI:** 10.3389/fonc.2014.00193

**Published:** 2014-07-25

**Authors:** Nora Berois, Eduardo Osinaga

**Affiliations:** ^1^Laboratorio de Glicobiología e Inmunología Tumoral, Institut Pasteur de Montevideo, Montevideo, Uruguay; ^2^Departamento de Inmunobiología, Facultad de Medicina, Universidad de la República, Montevideo, Uruguay

**Keywords:** neuroblastoma, glycosylation, gangliosides, polysialic acid, galectin-1, glycosyltransferases, immunotherapy

The glycosyltransferases B4GALNT3 and B4GALT3 were mistakenly analyzed as B4GALNT3 in this review. The Table 1 was changed and the text in the last paragraph of “Glycosyltransferases as tumor markers in NB patients” was modified as follows.

**Table 1:** **Glycosyltransferases as neuroblastoma (NB) tumor markers**.

Enzyme	Method/sample	Clinical significance	Reference
β1,4-*N*-acetylgalactosaminyltransferase (GD2 synthase)	ICC/bone marrow	Molecular marker of metastatic NB	*J Clin Oncol*; 4:363–369 (1986)
	RT-PCR ECL/bone marrow	Molecular marker of metastatic NB	Cancer; 92:924–931 (2001)
	RT-PCR ECL/bone marrow	Molecular marker of metastatic NB	Am J Pathol; 159:493–500 (2001)
	qRT-PCR/bone marrow	Marker for minimal residual disease	J Clin Oncol; 21:1087–1093 (2003)
	qRT-PCR/bone marrow-PB	Prognostic marker (poor outcome)	Int J Cancer; 123:2849–2855 (2008)
Sialyltransferase STX (ST8SiaII)	qRT-PCR/bone marrow	Molecular marker of metastatic NB	Int. Cancer; 119:152–156 (2006)
*N*-acetylglucosaminyltransferase V (GnT-V)	qRT-PCR/primary tumor	Prognostic marker (better outcome)	FEBS Lett; 580:627–632 (2006)
UDP-polypeptide GalNAc-transferase 13 (GalNAc-T13 – *GALNT13*)	RT-PCR/bone marrow	Molecular marker of metastatic NB	Clin Chem; 52:1701–1712 (2006)
UDP-polypeptide GalNAc-transferase 9 (GalNAc-T9 – *GALNT9*)	RT-PCR/primary tumor	Prognostic marker (better outcome)	Clin Chem; 59(1):225–233 (2013)
β1,3-*N*-acetylglucosaminyltransferase-3 (*B3GNT3*)	IHC/primary tumor	Prognostic marker (better outcome)	Cancer Sci; 104:1600–1608 (2013)
β1,4-*N*-acetylgalactosaminyltransferase 3 (*B4GALNT3*)	IHC/primary tumor	Prognostic marker (better outcome)	Am J Pathol; 179:1394–1404 (2011)
β-1,4-galactosyltransferase III (*B4GALT3*)	IHC/primary tumor	Prognostic marker (poor outcome)	Clin Cancer Res; 19:1705–1716 (2013)

*ICC, immunocytochemistry; RT-PCR, reverse transcriptase-polymerase chain reaction; ECL, electrochemiluminescence; PB, peripheral blood; IHC, immunohistochemistry*.

β1,4-*N*-acetylgalactosaminyltransferase III (*B4GALNT3*) cloned by Sato et al., has been described as expressed in stomach, colon, and testis (136). This enzyme can transfer GalNAc residues to non-reducing terminal GlcNAc-β leading to the synthesis of GalNAcβ1–4GlcNAc (also known as LacdiNAc or LDN), which is a unique terminal structure in the outer chain moieties of human *N*-glycans (137), and also in *O*-linked oligosaccharide structures (138). The largest amount of *B4GALNT3* transcripts were found in gastric tissues, followed by colon, testis, and adrenal glands (136). Gastric expression of *B4GALNT3* was found regulated by cellular differentiation (139). In the human colon, Huang et al. reported that *B4GALNT3* is up-regulated in primary tumors comparing with the normal mucosa (140). They performed *in vitro* and *in vivo* experiments showing that overexpression of this enzyme increases malignant phenotype of colon cancer cells, and these phenotypic changes are associated with enhanced integrin and mitogen-activated protein kinase (MAPK) signaling, suggesting that *B4GALNT3* may play a crucial role in promoting malignant behavior of colon cancer (140). The same research team has recently published that β-1,4-galactosyltransferase III (*B4GALT3*) overexpression in colorectal cancer cells suppressed cell migration, invasion, and adhesion, while *B4GALT3* knockdown enhanced malignant cell phenotypes promoting cell migration and invasion (141). Surprisingly, an opposite situation was found for both enzymes in NB. Firstly Hsu et al. communicated that *B4GALNT3* expression positively correlates with the differentiation status of NB, predicting a favorable prognosis for patients and suppressing the malignant phenotype in cell lines experiments via decreasing β1-integrin signaling (142). By contrast, β-1,4-galactosyltransferase III (*B4GALT3*) expression in NB tumors correlated with advanced clinical stage, unfavorable histology, and lower survival rate (143). In conclusion, *B4GALNT3* in NB seems a good prognostic marker, while *B4GALT3* was suggested as poor outcome marker. Further work is necessary to elucidate subjacent molecular mechanisms for these enzymes.

## Conflict of Interest Statement

The authors declare that the research was conducted in the absence of any commercial or financial relationships that could be construed as a potential conflict of interest.

